# Risk Perception of COVID-19 in Indonesia During the First Stage of the Pandemic

**DOI:** 10.3389/fpubh.2021.731459

**Published:** 2021-10-21

**Authors:** Mila Tejamaya, Baiduri Widanarko, Dadan Erwandi, Amelia Anggarawati Putri, Stevan D. A. M. Sunarno, I Made Ady Wirawan, Bina Kurniawan, Yahya Thamrin

**Affiliations:** ^1^Department of Occupational Health and Safety, Faculty of Public Health, Universitas Indonesia, Depok City, Indonesia; ^2^Department of Public Health and Preventive Medicine, Faculty of Medicine, Udayana University, Denpasar City, Indonesia; ^3^Department of Occupational Safety and Health, Faculty of Public Health, Diponegoro University, Semarang, Indonesia; ^4^Department of Occupational Safety and Health, Faculty of Public Health, Hasanuddin University, Makassar, Indonesia

**Keywords:** COVID-19, risk perception, Indonesia, risk tolerance, pandemic

## Abstract

Community perceptions of early-stage pandemics may have significant implications for subsequent disease control and management. Perceptions of COVID-19 among Indonesian citizens were assessed 2 months after the first reported case in the country. The study used an online survey tool, which was adapted from a standardized questionnaire for risk perception of an infectious disease outbreak. The questions of the survey involved respondents' perceived level of knowledge, preparedness, efficacy of control measures, newness, infectiousness, seriousness, motivating and hindering factors, and effectiveness of prevention methods, as well as questions that assessed actual level of knowledge of respondents such as causative agents, modes of transmission, number of total cases, and available control measures. A total of 1,043 respondents participated in this study. The main sources of information of respondents were social media (85.2%) and online news (82.2%). Nearly all respondents were aware that COVID-19 is a viral disease with saliva droplets (97.1%) and contaminated surfaces (86.5%) being its main modes of transmission. Participants showed a good level of knowledge pertaining to control measures, an adequate level of belief toward their efficacy, and a willingness to implement such measures. More than 95% of the respondents perceived COVID-19 to be either serious or very serious. However, the level of anxiety among respondents was moderate, suggesting the presence of risk tolerance in the community. Individual characteristics such as gender, educational background, and occupation were found to have a statistically significant relationship with risk perception and tolerance, but voluntary participation in control measures was high and similar. This indicates that the COVID-19 health campaign during early pandemic in Indonesia was a success. This research also revealed certain areas where health promotion, education, and awareness might be improved.

## Introduction

On December 31, 2020, China reported having found a cluster of new pneumonia cases in Wuhan, Hubei Province, caused by the SARS-CoV-2 virus (1). At that time, the average incubation period of COVID-19 was estimated to be 5 days (2). Due to the rapid transmission, on March 31, 2020, COVID-19 was declared as a pandemic (3). According to several studies conducted during the early stages of the COVID-19 pandemic, men were at a higher risk of developing severe health outcomes and had a higher fatality rate than women (4–6). With regard to the age of the victim, COVID-19 was found to be more fatal toward individuals of older age (7–9).

In order to reduce the transmission of COVID-19, behavioral changes in communities play a crucial role (10, 11). The transmission of a disease is influenced by behavioral response of an individual, such as adopting preventive measures, which is shaped by their perceptions (12–14). Managing public health risks, especially during the current COVID-19 pandemic, greatly relies on the ability of a community to appreciate those risks (15). Risk perception of an individual significantly influences their motivation to change their health behavior (16–18).

On the other hand, how a person perceives risk is not always associated with the epidemiological risk (19). Looking back at the Ebola outbreak in 2014, perception of the public toward a disease is influenced by their knowledge, which originates from their various information sources (20–22). Risk perceptions have been widely accepted as a main concept in navigating people toward achieving a suitable health behavior (23). At the same time, however, risk tolerance, a feeling of individual capability to control the risks, may lead to optimism bias and cause a person to become more relaxed toward an unsafe behavior (24). Hence, balancing the level of risk perception and risk tolerance is crucial in controlling risk (25).

Based on several theories (i.e., Protection Motivation Theory, Risk Compensation/Risk Homeostasis Theory, Situated Rationality Theory, Habituated Theory, Social Action Theory, and Social Control Theory), the Campbell Institute (25) created a model of factors that affects risk perception and risk tolerance. The various factors are categorized based on their scale, namely, macro-level (structural or institutional factors), meso-level (peer-to-peer or community factors), and micro-level (individual factors) (25).

On March 2, 2020, the first and second cases of COVID-19 were officially declared by Indonesian President, Joko Widodo. Following the announcement, an online platform was established for COVID-19-related communication between the Indonesian government and its citizens (www.covid19.go.id). Since then, risk communication from the Indonesian government has continued, not only *via* the website but also through television and other forms of public media. The main goal of risk communication by the Indonesian government during that stage was to update the total number of emerging COVID-19 cases and to suggest COVID-19 control measures, e.g., hand sanitizing, staying at home, etc. Whether the provided information and other sources of information have shaped risk perception and risk tolerance of Indonesian citizens is yet to be investigated.

Several “local” studies in Indonesia found low levels of anxiety and risk perception (26, 27). This study was aimed to assess the risk perception and risk tolerance among Indonesian citizens from a large study population, focusing on those who resided in the areas most affected by COVID-19 during the time of research. According to www.covid19.go.id, in early May 2020, the seven provinces that had the highest number of COVID-19 cases were Jakarta, West Java, Central Java, East Java, South Sulawesi, Bali, and Serang. Individual factors associated with perceived risk will be investigated to be able to provide meaningful recommendations for risk communicators and the government for optimizing health promotion to the public.

## Methods

### Instrument

Since most people were working at home during the COVID-19 pandemic, an online survey was conducted using a self-administered questionnaire. Indonesian residents aged 18 years or more were eligible to participate in this cross-sectional study. A link to the online questionnaire using a Google form was circulated among the potential participants *via* WhatsApp (Facebook) messenger application in contacts of the investigators. Snowball sampling methods were applied to gather potential participants. Ethics approval for the study was obtained from the Research and Community Engagement Ethical Committee, Faculty of Public Health Universitas Indonesia (164/UN2.F10.D11/PPM.00.02/2020).

A set of standardized questions sought information on demographics (i.e., gender, age, marital status, religion, job title, education background, city/town, and province of residence). Regarding COVID-19, a standardized self-administered questionnaire from ECOM (Effective Communication in Outbreak Management for Europe 2015) was used to gather information on knowledge (two questions), disease background (three questions), and risk perception (eight questions of COVID-19) downloaded from http://ecomeu.info/wp-content/uploads/2015/11/Standard-questionnaire-risk-perception-ECOM-november-2015.pdf

### Sample Size

The study population includes COVID-19-confirmed cases from all Indonesian provinces (34 provinces in total), which, on April 24, 2020, were 8,185 confirmed cases. Of 8,185 confirmed cases, 6,682 confirmed cases were from DKI Jakarta (3,599), West Java (862), East Java (690), Central Java (575), South Sulawesi (420), Banten (359), and Bali (177). By applying Slovin's formula (with assumption that there were 8,185 confirmed cases, 95%CI, and a margin of error of 5%), the minimum sample size for the present study was 368 participants. Based on the proportion of each province's confirmed cases relative to the total confirmed cases, the sample from each province is as follows: DKI Jakarta (198), West Java (28), East Java (29), Central Java (30), South Sulawesi (24), Banten (21), and Bali (10).


(1) $$\begin{array}{r} n=\;\frac{N}{1+Ne^{2}} \end{array}$$

### Data Analysis

The prevalence of each response for every question was calculated. Differences in prevalence among groups were assessed using the chi-square test. The level of significance was set at *p* < 0.05. All statistical analyses were conducted using Statistical Package for the Social Sciences version 23.0 [Statistical Package for the Social Sciences (SPSS) Statistics 23, 2014].

## Results

The survey results are described in the following paragraphs, and the associations between perceptions and sociodemographic variables are provided in Supplementary Materials.

### Characteristics of the Survey Respondents

A total of 1,043 respondents participated in this study, representing 30 out of the 34 total provinces in Indonesia with more than 90% of the respondents resided in the seven provinces mentioned (DKI Jakarta, West Java, East Java, Central Java, South Sulawesi, Banten, and Bali). Sociodemographic characteristics of the respondents are presented in Table 1. Out of the 1,043 respondents, 40.9% (*n* = 425) were male and 59.1% (*n* = 615) female, while three respondents preferred not to answer. The majority of the respondents (66.3%, *n* = 692) were 26–45 years old (adults). Based on their marital status, 63.9% (*n* = 666) of the respondents were married, and 36.1% (*n* = 377) were unmarried. According to their occupational status, most of the respondents were working as an employee at a private company (30.2%, *n* = 315) and as civil servants (24.4%, *n* = 254). Half of the respondents (55.2%, *n* = 576) were graduates, and 29.2% (*n* = 305) of postgraduates represent the educated group of Indonesian population.

**Table 1:** Sociodemographic characteristics of the respondents (*N* = 1,043).

**Characteristics**	**Participants**	
	***n***	%^*^
**Sex (*****n** **=*** **1,040)**		
Male	425	40.9
Female	615	59.1
Preferred not to answer	3	
**Age (*****n** **=*** **1,043)**		
(18–25)	230	22.1
(26–45)	692	66.3
(46–65)	117	11.2
(>65)	4	0.4
**Marital Status (*****n** **=*** **1,043)**		
Married	666	63.9
Unmarried	377	36.1
**Religion (*****n** **=*** **1,043)**		
Islam	748	71.7
Catholic	32	3.1
Christian	82	7.9
Buddha	4	0.4
Hindu	164	15.7
Preferred not to answer	13	1.2
**Occupation (*****n** **=*** **1,043)**		
Civil Servant	254	24.4
Private Company	315	30.2
Student	140	13.4
Housewife	117	11.2
Others	217	20.8
**Educational Background (*****n** **=*** **1,043)**		
Senior high school	162	15.5
Graduate	576	55.2
Post-graduate	305	29.2

* *Note: $\%=\;\frac{n}{N}$*.

### Level of Knowledge of COVID-19

Four choices were provided in the questionnaire pertaining to the respondent's level of knowledge of COVID-19, which were none, little, average, and above average. Of the 1,043 respondents, the majority (70.9%, *n* = 740) identified as having an average level of knowledge, 19.2% (*n* = 200) indicated that they had an above-average level of knowledge, while the remaining respondents identified as having very little (9.6%, *n* = 100) and none (0.3%, *n* = 3). There was a significant difference in respondents' perceived level of knowledge between males and females (*p* < 0.001) and between educational background groups (*p* = 0.000) (Supplementary Tables 1, 2).

### Source of Information

The respondents were asked where their sources of information originated from, in which they could answer more than one. The sources included online news, social media, television, newspaper, radio, word of mouth, and others. Interestingly, social media (85.2%) and online news (82.2%) were the two most accessed sources, followed by television (63%) and word of mouth (45.3%). However, <20% of the respondents retrieved information on COVID-19 from the newspaper (12.9%), radio (9.4%), and other sources (18.5%). Married respondents identified word of mouth (*p* = 0.042) and radio (*p* = 0.012) as a source of information more frequently than unmarried respondents (Supplementary Tables 1, 2).

### Disease Background Information

Understanding of respondents about the causative agent of COVID-19 and its modes of transmission was examined. The vast majority of the respondents (99.8%, *n* = 1,041) were aware that COVID-19 is a viral disease. Respondents were also asked about the knowledge of the modes of transmission, where the five options were saliva droplets, contaminated surfaces, food, water, and animal bites, and respondents could select more than one answer. The majority (97.1%) of respondents (*n* = 1,037) agreed that saliva droplets are the main route of transmission, followed by contaminated surfaces (86.5%, *n* = 921). Other modes such as contaminated food, water, and animal bites accounted for <20% each. Interestingly, those who have had contact with an active COVID-19 patient were more likely to perceive contaminated food (*p* < 0.01) and water (*p* < 0.01) as modes of transmission.

Participants were also asked about their knowledge of available COVID-19 control measures, where the choices included hand sanitizing, physical distancing, wearing a face mask, staying at home, exercising, and consuming nutritious food, in which respondents could choose one than one option. The vast majority believed that hand sanitizing (95.9%, *n* = 1,000), physical distancing (95.6%, *n* = 997), wearing a face mask (94.7%, *n* = 988), and staying at home (91.9%, *n* = 958) are effective in controlling COVID-19 infection. In addition, the respondents also believed that exercising (74.7%, *n* = 779) and consuming nutritious foods (87.6%, *n* = 914) are effective. There was a significant difference between females and males perceptions toward the effectivity control measures (*p* < 0.05). Educational background and occupation also affected the perception of respondents toward the efficacy of control measures, which can be seen in Supplementary Tables 3, 4.

### Perception of COVID-19

**Figure 1:**
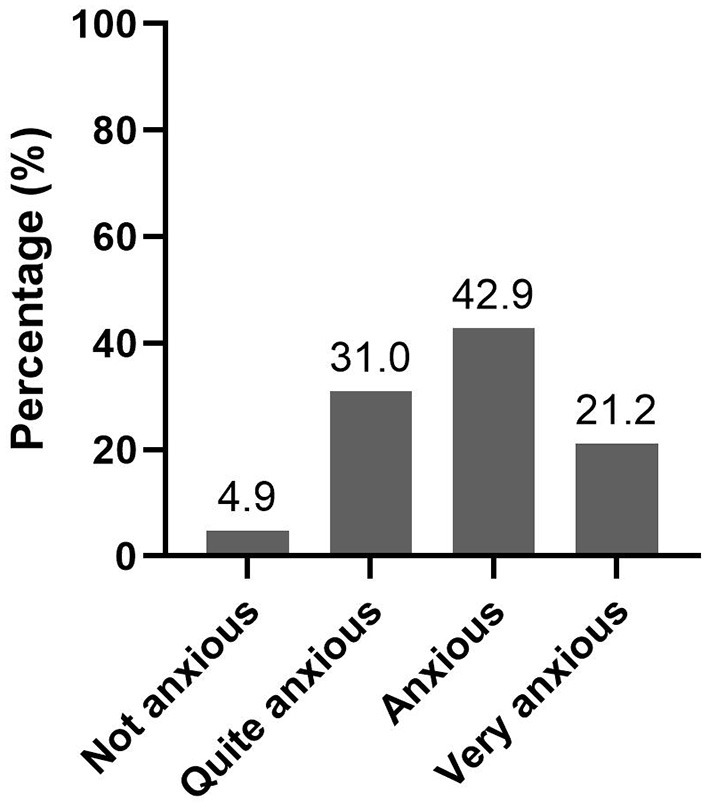
Level of anxiety toward COVID-19 in Indonesia (*n* = 1,043).

Interestingly, the level of anxiety due to COVID-19, in this study population, was densely distributed between quite anxious (31%, *n* = 323), anxious (43%, *n* = 448), and very anxious (21.4%, *n* = 223) (Figure 1). It can be concluded that the level of anxiety of respondents was moderate.

To explain these findings, the state of the risk perception and that of the risk tolerance of the respondents were examined in terms of the interaction between fear and anxiety.

**Figure 2:**
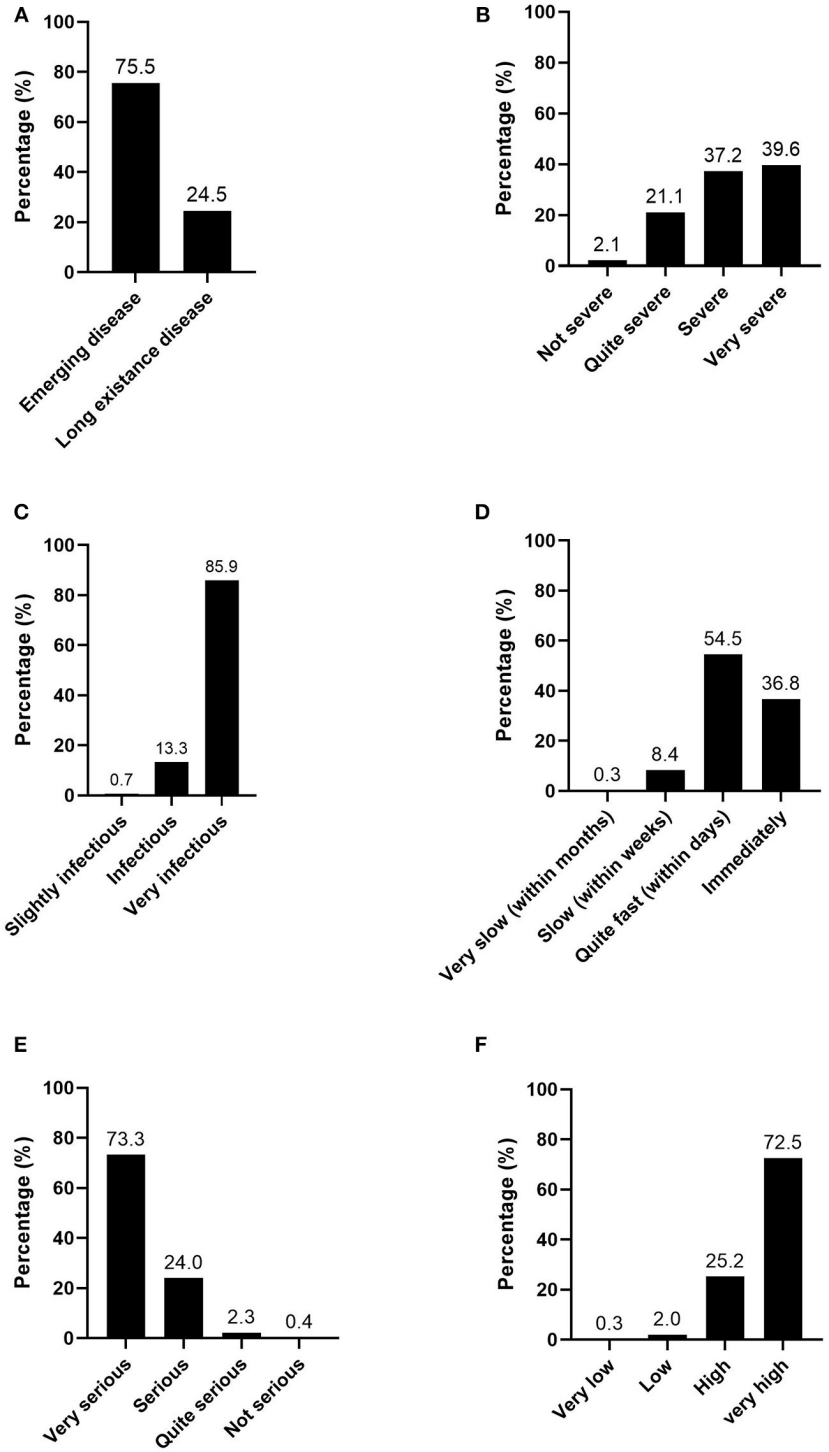
Perception on: **(A)** Newness; **(B)** Severity; **(C)** Infectiousness; **(D)** Contagiousness; **(E)** Seriousness; and **(F)** Total cases of COVID-19 in Indonesia (*n* = 1,043).

In this study, the risk perception of the respondents on COVID-19 was qualitatively assessed through the perceptions of the newness, severity, infectiousness, contagiousness, seriousness, and total cases of the disease of the respondents (Figure 2). In general, the respondents perceived COVID-19 as a high-risk disease that is emerging (75.8%, *n* = 791), and 77% (*n* = 803) believed that its severity ranges from severe to very severe. The majority (73.3%, *n* = 764) agreed that it is a very serious disease, that it spreads rapidly (within days to immediately) (91.4%, *n* = 953), and that the total number of cases varies between high and very high (98.7%, *n* = 1,020).

Risk perception is associated with various individual factors. In our study, the most influential sociodemographic factors were sex, occupation, and level of education (refer to Supplementary Tables 5, 6).

**Figure 3:**
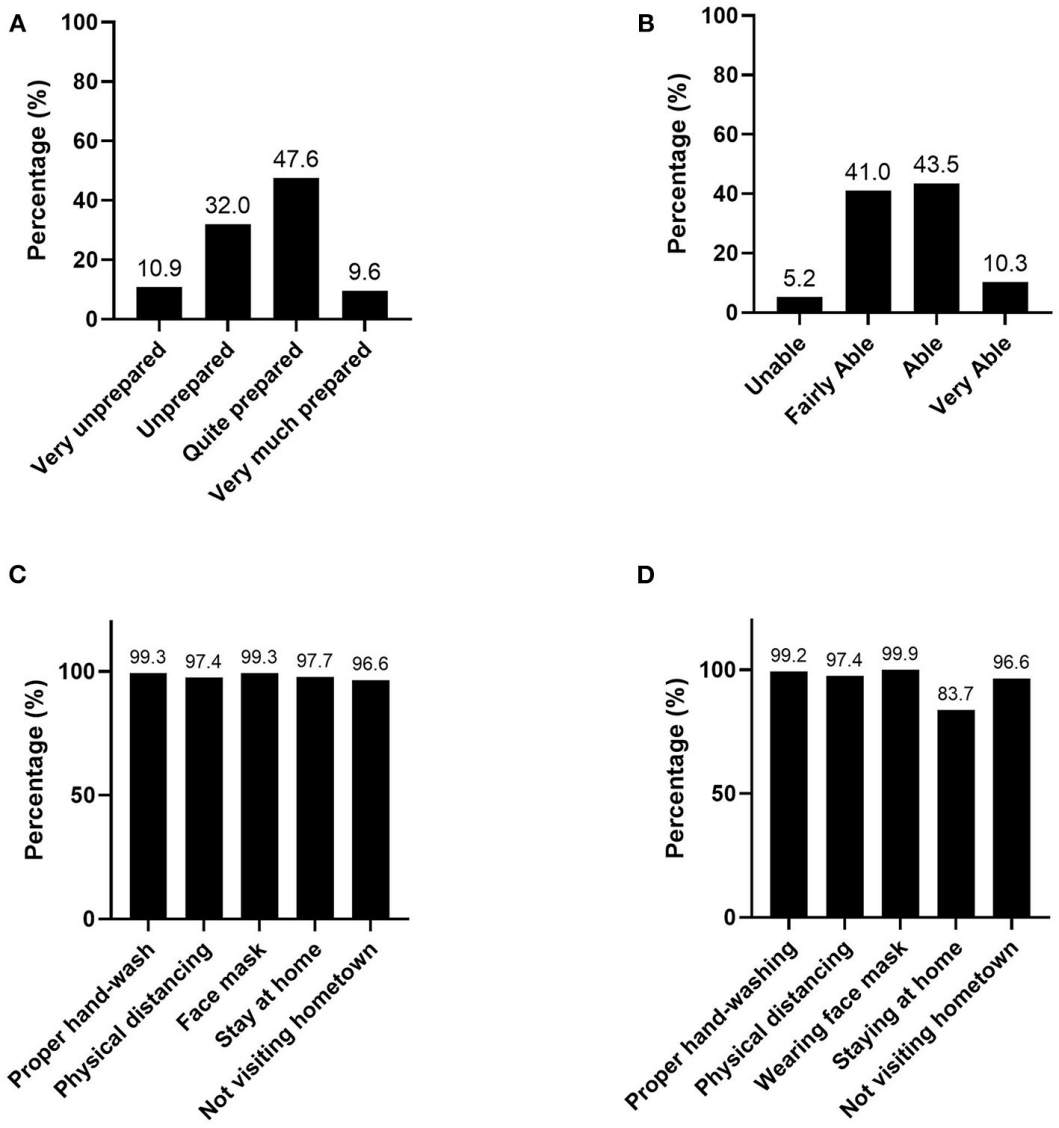
Perception on: **(A)** Preparedness; **(B)** Ability to control the risk; **(C)** Efficacy of Control Measures; and **(D)** Willingness to carry out measures of COVID-19 in Indonesia (*n* = 1,043).

Conversely, we found that the level of risk tolerance of respondents ranged from moderate to high (Figure 3). Almost half (47.4%) of the respondents perceived that they were quite prepared to face COVID-19. They perceived their ability to control COVID-19 risks to be “fairly able” (41.1%) to “able” (43.4%). Most strikingly, more than 97% were willing to perform hand sanitizing, physical distancing, and wearing a face mask. Though 97% agreed that staying at home is important, only 83.4% would carry out this control due to job requirements.

It is important to note that during the course of this study, the Eid holiday period was quickly approaching. The Government emphasized the notion that if those who reside in the metropolitan areas were to visit their hometown, as is the norm here during this time of year, they could infect those back home. Interestingly, this message was well accepted by the people with 96.6%, indicating that they would refrain from visiting their hometown. This study classifies this action as a control measure as it follows the same basic principle as social distancing. As is the case with risk perception, risk tolerance is also associated with sociodemographic variables (Supplementary Tables 7, 8).

### Motivating and Hindering Factors in Carrying Out Control Measures

**Figure 4:**
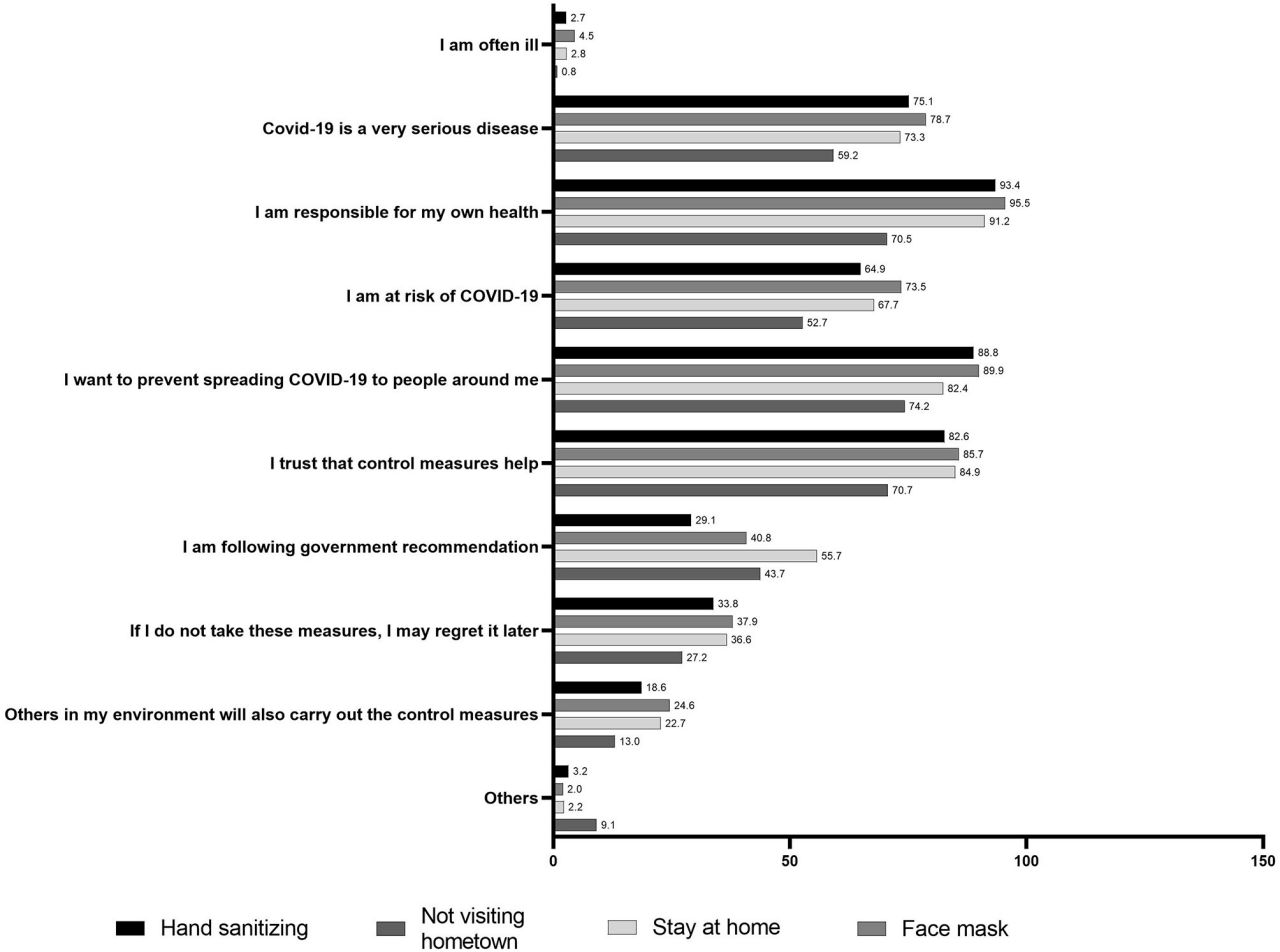
Motivating factors that drive the willingness to carry out preventive measures of COVID-19 in Indonesia (*n* = 1,043).

Motivation is crucial to drive the implementation of control measures. This study found that the main motivating factor for implementing control measures, apart from “not visiting hometown during Eid,” was their sense of responsibility toward their own health, followed by their desire to avoid spreading the virus to others and their trust in the efficacy of the control measures (above 82%)—(see Figure 4). The cause of why the respondents would not visit their hometown was likely due to their sense of responsibility to protect health of others, as promoted by the Government. The most dominant hindering factor, however, is caused by the lack of facilities, e.g., not enough public faucets, not having face masks, etc., followed by peer pressure from those who do not carry out the measures (Table 2).

**Table 2:** Hindering factors that hinder the willingness to carry out preventive measures of COVID-19 in Indonesia.

**Hindering factors**	**Hand sanitizing (*N* = 8)**		**Physical distancing (*N* = 28)**		**Wearing a face mask (*N* = 1)**		**Staying at home (*N* = 174)**		**Not visiting hometown (*N* = 36)**	
	***n***	%^*^	***n***	%^*^	***n***	%^*^	***n***	%^*^	***n***	%^*^
Absence of face mask	NA	NA	NA	NA	1	100	NA	NA	NA	NA
Hard to find hand-washing facilities	4	50	NA	NA	NA	NA	NA	NA	NA	NA
I am never ill	0	0	1	3.6	0	0	2	1.1	2	5.6
COVID-19 is not a serious disease	0	0	0	0	0	0	0	0	0	0
I am not worried about my health	0	0	1	3.6	0	0	2	1.1	2	5.6
I do not think I am at risk of contracting COVID-19	0	0	1	3.6	0	0	1	0.6	5	13.9
I do not think that I would spread COVID-19 to others	0	0	1	3.6	0	0	6	3.4	7	19.4
I doubt that control measure will help	1	12.5	7	25.0	0	0	1	0.6	2	5.6
Takes too much effort	0	0	9	32.1	0	0	0	0	0	0
I feel that too little information is provided about the control measure	0	0	5	17.9	0	0	0	0	1	2.8
People in my environment will also not carry out the measures	0	0	13	46.4	0	0	14	8.0	2	5.6
My immediate family is living at hometown	NA	NA	NA	NA	NA	NA	NA	NA	15	41.7
My extended family is living at hometown	NA	NA	NA	NA	NA	NA	NA	NA	21	58.3
Job requirement	NA	NA	NA	NA	NA	NA	155	89.1	NA	NA
Financial cause	NA	NA	NA	NA	NA	NA	35	20.1	4	11.1
Others	3	37.5	10	35.7	0	0	24	13.8	9	25.0

* *Note: $\%=\;\frac{n}{N}$*.

## Discussion

### Level of Knowledge and Source of Information

The findings of this study indicate that most of our respondents believed that their knowledge pertaining to COVID-19 is satisfactory. Almost 100% of the respondents understood that the causative agent of COVID-19 is a virus that can be transmitted *via* saliva droplets (97.1%) and contaminated surfaces (86.5%); this can serve as an indication that the respondents of this study had an adequate level of knowledge regarding COVID-19. Two individual factors had a statistically significant association with respondents' level of knowledge of COVID-19, namely, sex (*p* < 0.001) and level of education (*p* < 0.001).

Though an association between level of education and knowledge on COVID-19 makes logical sense, interestingly, there is a statistically significant association between knowledge and gender. While most female respondents perceive their knowledge on COVID-19 to be average, more male respondents identified as above average. Contrary to their perception, in reality, female respondents were found to be more knowledgeable on available control measures, i.e., proper hand washing, wearing a face mask, physical distancing, staying at home, exercising at home, and consuming nutritious food (*p*-value ranges from < 0.001 to 0.044).

Previous studies have found gender to be an important determinant of health, such as that in 2000, wherein this statement was acknowledged by the WHO (31). Other studies have shown that women tend to implement more preventive and health behavior compared to men (32, 33). Since health behaviors can be linked with gender, effective health promotion and communication need to be more specialized toward its target demographic, which the WHO calls gender approach (report WHO) (34).

In terms of source of information regarding COVID-19, online news, social media, and television were the most accessed sources of information in our study population. Other sources of information such as word of mouth, newspaper, and radio were accessed less than their online counterparts. These findings highlight the significance of online news, social media, and television as important means of risk communication and health promotion in Indonesia, and social media should be directed to support public health promotion (35). This finding is consistent with the study from Wang et al. in Taiwan who found that information from the internet was the most frequently accessed COVID-19 information (30). At quite the same time, in Jordan, Olaimat et al. revealed that the most of the university students relied on internet and social media as source of information regarding COVID-19 (36). Yet, in all states of the US, Ali et al. discovered that most of US citizens access to traditional media as source of information regarding COVID-19 (37).

However, the downside of having online news, social media, and even television in Indonesia is the spread of misinformation or hoax. As indicated by low index of uncertainty avoidance culture according to Hofstede's theory (38), Indonesian is more tolerant to misinformation compared to high uncertainty avoidance culture (39). Much of the misinformation or disinformation feeds into fears of government control, conspiracies, and distrust of vaccination. Certain Indonesian TV channels are directly linked to various political parties, which may result in biased reporting. Unfortunately, it is very difficult to eliminate such things from happening due to its massive scale. Bridgman et al. and Pulido et al. found that misinformation may hinder the adoption of pandemic control measures and intensify the pandemic due to opportunity of misinformation to be shared between individual was more frequently than science-based evidence or public health recommendations (29, 40). Thus, Indonesian government and risk communicators should be aware of potential hoax news and clarify that misinformation through online platform since social media and online news has been claimed as the most accessed source of information regarding COVID-19.

### Risk Perception and Risk Tolerance

It was found that almost all respondents believed COVID-19 to be an emerging, infectious, serious, contagious disease and that there was a high number of total confirmed cases. Fortunately, respondents also believed that they were moderately capable of controlling the risks as they believed in the efficacy of the promoted control measures and were willing to implement them. A survey conducted in Indonesia by Lembaga Demografi (41) found similar results. Even though their sociodemographic variables differed from our study, it was found that Gen Z (those born between 1995 and 2012) individuals who actively use Instagram agree that COVID-19 is dangerous and contagious; however, the majority of the respondents believe in the efficacy of health protocols (wearing face mask and face shield, hand sanitizing, and social distancing) and 75% of them were willing to implement those controls (41). According to Extended Parallel Processing Model (EPPM), high perception on both threat and efficacy will lead to individual practice on self-protective behavior (42, 43) as high coping capability by Bandura (44). As shown in the supplementary materials (Supplementary Tables 3, 4), various individual factors had statistically significant associations with risk perception and tolerance; however, willingness to implement COVID-19 control measures, which include hand washing, physical distance, wearing a facemask, and staying at home, was quite similar. This data could imply that a health protocol campaign is in the works.

Self-responsibility in protecting own health of an individual became the main motivation that drove the willingness of respondents to control the associated risks. However, the absence or inaccessibility of facilities such as hand washing faucets and face masks, together with peer pressure, were perceived as hindering factors in performing health behaviors.

Perceived efficacy and adoption of control measures from this study were comparable with the result conducted in Hong Kong by Kwok et al. who reported that 96.7 and 94.9% of the respondents believed the efficacy of frequent hand washing and wearing a face mask, respectively (45). At lesser amount, only 81.6% of Kwok's study's participants had confidence in the efficacy of staying at home. Lower risk tolerances were shown in two studies: one conducted in three Middle Eastern countries and the other in Myanmar. According to the Middle Eastern study conducted by Shahin and Hussein, the willingness among their respondents to perform handwashing, wearing a face mask, and social distancing was only 66.8, 61.0, and 67.6%, accordingly (46). From the study conducted in Myanmar, Mya et al. found that 84% of their respondents believed in the efficacy of washing hands and 72% believed in the efficacy of wearing a face mask (47).

Balancing the level of risk perception and the level of risk tolerance is crucial in controlling risk and negative emotions (25, 48). Due to comparable levels of risk perception and risk tolerance in this study, most respondents had an appropriate level of “fear” with the perceived level of anxiety being distributed in the middle, ranging from quite anxious to anxious. Only 21.4% of the respondents were classified as very anxious, and 4.7% were not anxious at all. Several individual factors were associated with respondents' level of anxiety, namely, gender, age, marital status, and occupation.

It was shown in this study that married women and housewives had a higher level of anxiety in contrast to other groups of respondents. This might be partially associated by their concern for the health of loved ones as highlighted in the study by Mertens et al. (49). Hou et al. also found that women in China showed greater a extent of anxiety due to COVID-19 (28). Heffner et al. revealed that gender, anxiety, and social media exposure may increase the vulnerability of negative distress (50). Age can also be another predictor of anxiety. In this study, late adults (46–65 years old) also showed a higher level of anxiety, which may be acquitted to the fact that, during the early stages of the pandemic in Indonesia, fatality rate among the older population was higher, despite confirmed cases being very prevalent among the younger population. This study identified potential vulnerable groups of the Indonesian population who are prone to the various negative emotional impacts caused by COVID-19, hence why they require extra protection, specifically regarding their mental health.

The limitations of this study include the following: (1) the study population was dominated by the highly educated part of the population and (2) the study population was almost exclusively from only seven provinces, consistent with the aims of this study. Therefore, generalization of findings of this study to the national population should be interpreted in context.

## Conclusion

In conclusion, among the educated section of population of Indonesia, it was discovered that they consider their degree of knowledge on COVID-19 to be adequate. A high level of risk perception was counterbalanced by a high level of tolerance and voluntariness in putting control mechanisms in place. This finding suggests that the health protocol campaign in Indonesia during the early stages of the pandemic was a success. This study also discovered certain areas where health education, promotion, and campaigning may be improved, particularly in the area of mental health protection.

## Data Availability Statement

The original contributions presented in the study are included in the article/Supplementary Material, further inquiries can be directed to the corresponding author/s.

## Ethics Statement

The studies involving human participants were reviewed and approved by ethical approval for this study was obtained from the Research and Community Engagement Ethical Committee. Faculty of Public Health. Universitas Indonesia (Ethical Clearance Number: Ket-164/UN2.F10.D11/PPM.00.02/2020). The patients/participants provided their written informed consent to participate in this study.

## Author Contributions

MT contributed to the process of research proposal preparation, data collection and analysis, and manuscript writing. BW and DE contributed to the research proposal preparation, data collection, and analysis. AP and SS contributed to data collection and analysis. IW contributed to proposal preparation, data collection, and analysis. BK and YT contributed to data collection. All authors contributed to the article and approved the submitted version.

## Conflict of Interest

The authors declare that the research was conducted in the absence of any commercial or financial relationships that could be construed as a potential conflict of interest.

## Publisher's Note

All claims expressed in this article are solely those of the authors and do not necessarily represent those of their affiliated organizations, or those of the publisher, the editors and the reviewers. Any product that may be evaluated in this article, or claim that may be made by its manufacturer, is not guaranteed or endorsed by the publisher.
