# Supplementary material for: Risk Perception of COVID-19 in Indonesia During the First Stage of the Pandemic
Source: Front Public Health. 2021 Oct 21;9:731459. doi: 10.3389/fpubh.2021.731459 (PMC8566751; doi:10.3389/fpubh.2021.731459)
Supplement: Supplementary file 1 [file Data_Sheet_1.pdf]

Supplementary Materials

Risk Perception of COVID-19 in Indonesia During the First Stage of the Pandemic

Mila Tejamaya[1,*], Baiduri Widanarko[1], Dadan Erwandi[1], Amelia Anggarawati[1], Stevan D.A.M. Sunarno[1], I Md Ady Wirawan[2], Bina Kurniawan[3], Yahya Thamrin[4]

[1]*Department of Occupational Health and Safety, Faculty of Public Health, Universitas Indonesia, Depok City, West Java Province, Indonesia*
[2]*Department of Public Health and Preventive Medicine, Faculty of Medicine, Udayana University, Denpasar City, Bali Province, Indonesia*
[3]*Department of Occupational Health and Safety, Faculty of Public Health, Diponegoro University, Semarang City, Central Java Province, Indonesia*
[4]*Department of Occupational Health and Safety, Faculty of Public Health, Hasanuddin University, Makassar City, South Sulawesi Province, Indonesia*

* Author to whom correspondence should be addressed: tejamaya@ui.ac.id

Tabel S1. Association between Knowledge on COVID-19 with Sex, Age, Marital Status and Education (N=1043; %)

	Risk Perception Key Elements	Total	Sex			Age					Marital Status			Education			
			Male	Female	P-value	Early-Late Teens	Early-Late Adults	Early-Late Old	Elderly	P-value	Married	Unmarried	P-value	<=High School	Under Graduate	Graduate	P-value
A.	Knowledge on COVID-19																
1	Level of knowledge on Covid-19				<0.001					0.63			0.462				<0.001
	None	3 (0.3)	2 (0.5)	1 (0.2)		0 (0.0)	2 (0.3)	1 (0.9)	0 (0.0)		3 (0.5)	0 (0.0)		0 (0.0)	2 (0.3)	1 (0.3)	
	Little	100 (9.6)	58 (13.6)	42 (6.8)		21 (9.1)	64 (9.2)	15 (12.8)	0 (0.0)		68 (10.2)	32 (8.5)		28 (17.3)	50 (8.7)	22 (7.2)	
	Average	740 (70.9)	256 (60.2)	481 (78.2)		166 (72.2)	496 (71.7)	76 (65.0)	2 (50.0)		468 (70.3)	272 (72.1)		113 (69.8)	427 (74.1)	200 (65.6)	
	Above Average	200 (19.2)	109 (25.6)	91 (14.8)		43 (18.7)	130 (18.8)	25 (21.4)	2 (50.0)		127 (19.1)	73 (19.4)		21 (13.0)	97 (16.8)	82 (26.9)	
2	Source of Information (can choose more than one answer)																
	Online news	857 (82.2)	343 (80.7)	511 (83.1)	0.366	191 (83)	566 (81.8)	96 (82.1)	4 (100)	0.788	549 (82.4)	308 (81.7)	0.831	135 (83.3)	473 (82.1)	249 (81.6)	0.901
	Social media	889 (85.2)	358 (84.2)	529 (86)	0.479	201 (87.4)	581 (84)	103 (88)	4 (100)	0.367	558 (83.8)	331 (87.8)	0.096	138 (85.2)	492 (85.4)	259 (84.9)	0.980
	Television	657 (63.0)	277 (65.2)	378 (61.5)	0.249	141 (61.3)	434 (62.7)	78 (66.7)	4 (100)	0.343	414 (62.2)	243 (64.5)	0.503	101 (62.3)	356 (61.8)	200 (65.6)	0.536
	Newspaper	135 (12.9)	56 (13.2)	78 (12.7)	0.889	27 (11.7)	90 (13.0)	17 (14.5)	1 (25.0)	0.783	90 (13.5)	45 (11.9)	0.527	18 (11.1)	80 (13.9)	37 (12.1)	0.572
	Radio	98 (9.4)	37 (8.7)	60 (9.8)	0.643	19 (8.3)	71 (10.3)	8 (6.8)	0 (0.0)	0.518	66 (9.9)	32 (8.5)	0.012	6 (3.7)	66 (11.5)	26 (8.5)	0.009
	Word of mouth	473 (45.3)	196 (46.1)	275 (44.7)	0.702	91 (39.6)	329 (47.5)	52 (44.4)	1 (25.0)	0.161	322 (48.3)	151 (40.1)	0.042	72 (44.4)	262 (45.5)	139 (45.6)	0.968
	Others	193 (18.5)	93 (21.9)	100 (16.3)	0.027	35 (15.2)	135 (19.5)	23 (19.7)	0 (0.0)	0.373	136 (20.4)	57 (15.1)	0.078	24 (14.8)	107 (18.6)	62 (20.3)	0.344

Tabel S2. Association between Knowledge on COVID-19 with Physical Contact with Covid-19 patient, Occupation and Residence (N=1043; %)

Risk Perception Key Elements		Total	Physical contact with COVID-19 patient				Occupation						Residence								
			**Yes**	**No**	**Not sure**	**P-value**	**Civil Servant**	**Private Company**	**Student**	**Housewife**	**Others**	**P-value**	**Jakarta**	**West Java**	**East Java**	**Central Java**	**South Sulawesi**	**Banten**	**Bali**	**Others**	**P-value**
A. Knowledge on COVID-19																					
1	Level of knowledge on Covid-19					0.858						0.224									0.425
	None	3 (0.3)	0 (0.0)	3 (0.3)	0 (0.0)		1 (0.4)	1 (0.3)	0 (0.0)	0 (0.0)	1 (0.5)		0 (0.0)	0 (0.0)	0 (0.0)	0 (0.0)	1 (1.3)	0 (0.0)	2 (1.1)	0 (0.0)	
	Little	100 (9.6)	4 (10.5)	79 (9.2)	17 (11.7)		28 (11.0)	32 (10.2)	10 (7.1)	8 6.8)	22 (10.1)		16 (7.9)	21 (9.9)	1 (2.6)	17 (10.3)	8 (10.3)	4 (6)	24 (13.4)	9 (8.9)	
	Average	740 (70.9)	26 (68.4)	609 (70.8)	105 (72.4)		186 (73.2)	209 (66.3)	103 (73.6)	95 (81.2)	147 (67.7)		147 (72.4)	146 (68.9)	30 (78.9)	113 (68.5)	52 (66.7)	50 (74.6)	130 (72.6)	72 (71.3)	
	Above Average	200 (19.2)	8 (21.1)	169 (19.7)	23 (15.9)		39 (15.4)	73 (23.2)	27 (19.3)	14 (12.0)	47 (21.7)		40 (19.7)	45 (21.2)	7 (18.4)	35 (21.2)	17 (21.8)	13(19.4)	23 (12.8)	20 (19.8)	
2	Source of Information (can choose more than one answer)																				
	Online news	857 (82.2)	31 (81.6)	714 (83)	112 (77.2)	0.242	209 (82.3)	252 (80.0)	117 (83.6)	96 (82.1)	183 (84.3)	0.755	159 (78.3)	177 (83.5)	34 (89.5)	133 (80.6)	63 (80.8)	54 (80.6)	151 (84.4)	86 (85.1)	0.615
	Social media	889 (85.2)	35 (92.1)	730 (84.9)	124 (85.5)	0.468	222 (87.4)	268 (85.1)	121 (86.4)	99 (84.6)	179 (82.5)	0.654	166 (81.8)	177 (83.5)	34 (89.5)	147 (89.1)	66 (84.6)	57 (85.1)	154 (86)	88 (87.1)	0.618
	Television	657 (63.0)	26 (68.4)	541 (62.9)	90 (62.1)	0.765	148 (58.3)	201 (63.8)	88 (62.9)	73 (62.4)	147 (67.7)	0.326	122 (60.1)	147 (69.3)	27 (71.1)	95 (57.6)	52 (66.7)	39 (58.2)	112 (62.6)	63 (62.4)	0.277
	Newspaper	135 (12.9)	5 (13.2)	115 (13.4)	15 (10.3)	0.603	36 (14.2)	38 (12.1)	19 (13.6)	11 (9.4)	31 (14.3)	0.689	20 (9.9)	27 (12.7)	4 (10.5)	20 (12.1)	15 (19.2)	9 (13.4)	28 (15.6)	12 (11.9)	0.536
	Radio	98 (9.4)	1 (2.6)	87 (10.1)	10 (6.9)	0.163	26 (10.2)	31 (9.8)	11 (7.9)	9 (7.7)	21 (9.7)	0.895	14 (6.9)	16 (7.5)	5 (13.2)	18 (10.9)	8 (10.3)	3 (4.5)	26 (14.5)	8 (7.9)	0.131
	Word of mouth	473 (45.3)	14 (36.8)	391 (45.5)	68 (46.9)	0.534	130 (51.2)	142 (45.1)	57 (40.7)	52 (44.4)	92 (42.4)	0.239	81 (39.9)	103 (48.6)	16 (42.1)	86 (52.1)	41 (52.6)	25 (37.3)	85 (47.5)	36 (35.6)	0.050
	Others	193 (18.5)	8 (21.1)	161 (18.7)	24 (16.6)	0.757	48 (18.9)	68 (21.6)	17 (12.1)	21 (17.9)	39 (18.0)	0.212	159 (78.3)	177 (83.5)	34 (89.5)	133 (80.6)	63 (80.8)	54 (80.6)	151 (84.4)	86 (85.1)	0.615

Table S3. Association Between Disease Background Information and Sex, Age, Marital Status And Education (N=1043; %)

	Risk Perception Key Elements	Total	Sex			Age					Marital Status			Education			
			Male N (%)	Female	P-value	Early-Late Teens	Early-Late Adults	Early-Late Old	Elderly	P-value	Married	Unmarried	P-value	<=High School	Under Graduate	Graduate	P-value
Disease Background Information																	
1	Causative Agent				0.794					0.069			0.130				0.345
	Virus	1041 (99.8)	424 (99.8)	614 (99.8)		228 (99.1)	692 (100)	117 (100)	4 (100)		666 (100)	375 (99.5)		161 (99.4)	575 (99.8)	305 (100)	
	Bacteria	2 (0.2)	1 (0.2)	1 (0.2)		2 (0.9)	0 (0.0)	0 (0.0)	0 (0.0)		0 (0.0)	2 (0.5)		1 (0.6)	1 (0.2)	0 (0.0)	
2	Mode of Transmission																
	Saliva droplet	1013 (97.1)	415 (97.6)	595 (96.7)	0.389	212 (92.2)	680 (98.3)	117 (100)	4 (100)	<0.001	659 (98.9)	354 (93.9)	<0.001	152 (93.8)	560 (97.2)	301 (98.7)	0.011
	Contaminated surfaces	902 (86.5)	364 (85.6)	535 (87)	0.534	199 (86.5)	605 (87.4)	95 (81.2)	3 (75.0)	0.287	573 (86.0)	329 (87.3)	0.642	141 (87.0)	501 (87.0)	260 (85.2)	0.755
	Food-borne	185 (17.7)	86 (20.2)	99 (16.1)	0.088	44 (19.1)	124 (17.9)	16 (13.7)	1 (25.0)	0.617	116 (17.4)	69 (18.3)	0.783	27 (16.7)	111 (19.3)	47 (15.4)	0.335
	Water-borne	117 (11.2)	58 (13.6)	59 (9.6)	0.043	26 (11.3)	79 (11.4)	12 (10.3)	0 (0.0)	0.887	76 (11.4)	41 (10.9)	0.872	21 (13.0)	67 (11.6)	29 (9.5)	0.475
	Animal bites	49 (4.7)	24 (5.6)	25 (4.1)	0.240	14 (6.1)	28 (4)	7 (6)	0 (0.0)	0.517	27 (4.1)	22 (5.8)	0.249	9 (5.6)	25 (4.3)	15 (4.9)	0.793
3	Available Control Measures																
	Proper hand-washing	1000 (95.9)	401 (94.4)	596 (96.9)	0.044	223 (97.0)	661 (95.5)	112 (95.7)	4 (100)	0.782	637 (95.6)	363 (96.3)	0.735	155 (95.7)	554 (96.2)	291 (95.4)	0.853
	Wearing a face mask	988 (94.7)	395 (92.9)	590 (95.9)	0.036	214 (93.0)	658 (95.1)	112 (95.7)	4 (100)	0.585	632 (94.9)	356 (94.4)	0.858	148 (91.4)	545 (94.6)	295 (96.7)	0.047
	Physical distancing	997 (95.6)	395 (92.9)	599 (97.4)	0.001	221 (96.1)	659 (95.2)	113 (96.6)	4 (100)	0.849	633 (95)	364 (96.6)	0.326	153 (94.4)	550 (95.5)	294 (96.4)	0.611
	Staying at home	958 (91.9)	372 (87.5)	583 (94.8)	<0.001	209 (90.9)	631 (91.2)	114 (97.4)	4 (100)	0.115	617 (92.6)	341 (90.5)	0.261	147 (90.7)	521 (90.5)	290 (95.1)	0.049
	Exercise indoor at home	779 (74.7)	298 (70.1)	481 (78.2)	0.003	169 (73.5)	522 (75.4)	84 (71.8)	4 (100)	0.521	500 (75.1)	279 (74)	0.758	106 (65.4)	433 (75.2)	240 (78.7)	0.007
	Consume nutritious food	914 (87.6)	355 (83.5)	559 (90.9)	<0.001	201 (87.4)	611 (88.3)	98 (83.8)	4 (100)	0.480	588 (88.3)	326 (86.5)	0.448	133 (82.1)	509 (88.4)	272 (89.2)	0.063

Table S4. Association Between Disease Background Information and Physical Contact with Covid-19 patient, Occupation and Residence (N=1043; %)

Risk Perception Key Elements		Total	Physical contact with COVID-19 patient				Occupation						Residence								
			Yes	No	Not sure	P-value	Civil Servant	Private Company	Student	Housewife	Others	P-value	Jakarta	West Java	East Java	Central Java	South Sulawesi	Banten	Bali	Others	P-value
Disease Background Information																					
1	Causative Agent					0.332						0.724									0.836
	Virus	1041 (99.8)	38 (100)	859 (99.9)	144 (99.3)		254 (100)	314 (99.7)	140 (100)	117 (100)	216 (99.5)		202 (99.5)	212 (100)	38 (100)	165 (100)	78 (100)	67 (100)	178 (99.4)	101 (100)	
	Bacteria	2 (0.2)	0 (0.0)	1 (0.1)	1 (0.7)		0 (0.0)	1 (0.3)	0 (0.0)	0 (0.0)	1 (0.5)		1 (0.5)	0 (0.0)	0 (0.0)	0 (0.0)	0 (0.0)	0 (0.0)	1 (0.6)	0 (0.0)	
2	Mode of Transmission																				
	Saliva droplet	1013 (97.1)	38 (100)	832 (96.7)	143 (98.6)	0.255	253 (99.6)	312 (99)	130 (92.9)	113 (96.6)	205 (94.5)	<0.001	197 (97)	208 (98.1)	38 (100)	160 (97)	75 (96.2)	66 (98.5)	169 (94.4)	100 (99)	0.283
	Contaminated surfaces	902 (86.5)	32 (84.2)	743 (86.4)	127 (87.6)	0.850	225 (88.6)	264 (83.8)	122 (87.1)	108 (92.3)	183 (84.3)	0.126	175 (86.2)	185 (87.3)	34 (89.5)	141 (85.5)	71 (91.0)	57 (85.1)	151 (84.4)	88 (87.1)	0.904
	Food-borne	185 (17.7)	10 (26.3)	138 (16.0)	37 (25.5)	0.008	40 (15.7)	67 (21.3)	25 (17.9)	15 (12.8)	38 (17.5)	0.255	40 (19.7)	42 (19.8)	10 (26.3)	24 (14.5)	13 (16.7)	9 (13.4)	32 (17.9)	15 (14.9)	0.572
	Water-borne	117 (11.2)	8 (21.1)	84 (9.8)	25 (17.2)	0.005	27 (10.6)	35 (11.1)	15 (10.7)	11 (9.4)	29 (13.4)	0.823	21 (10.3)	20 (9.4)	5 (13.2)	20 (12.1)	4 (5.1)	8 (11.9)	23 (12.8)	16 (15.8)	0.461
	Animal bites	49 (4.7)	3 (7.9)	35 (4.1)	11 (7.6)	0.115	10 (3.9)	15 (4.8)	5 (3.6)	6 (5.1)	13 (6.0)	0.811	14 (6.9)	9 (4.2)	2 (5.3)	8 (4.8)	3 (3.8)	1 (1.5)	8 (4.5)	4 (4.0)	0.764
3	Available Control Measures																				
	Proper hand-washing	1000 (95.9)	36 (94.7)	826 (96)	138 (95.2)	0.831	244 (96.1)	304 (96.5)	135 (96.4)	111 (94.9)	206 (94.9)	0.872	196 (96.6)	208 (98.1)	37 (97.4)	154 (93.3)	74 (94.9)	65 (97.0)	168 (93.9)	98 (97.0)	0.295
	Wearing a face mask	988 (94.7)	35 (92.1)	813 (94.5)	140 (96.6)	0.460	247 (97.2)	298 (94.6)	129 (92.1)	110 (94)	204 (94.0)	0.245	192 (94.6)	204 (96.2)	36 (94.7)	149 (90.3)	74 (94.9)	64 (95.5)	173 (96.6)	96 (95.0)	0.263
	Physical distancing	997 (95.6)	34 (89.5)	824 (95.8)	139 (95.9)	0.174	247 (97.2)	300 (95.2)	136 (97.1)	112 (95.7)	202 (93.1)	0.217	194 (95.6)	206 (97.2)	37 (97.4)	154 (93.3)	75 (96.2)	64 (95.5)	168 (93.9)	99 (98.0)	0.507
	Staying at home	958 (91.9)	36 (94.7)	788 (91.6)	134 (92.4)	0.763	239 (94.1)	282 (89.5)	132 (94.3)	114 (97.4)	191 (88.0)	0.007	182 (89.7)	201 (94.8)	36 (94.7)	151 (91.5)	73 (93.6)	60 (89.6)	163 (91.1)	92 (91.1)	0.629
	Exercise indoor at home	779 (74.7)	32 (84.2)	640 (74.4)	107 (73.8)	0.383	207 (81.5)	222 (70.5)	101 (72.1)	89 (76.1)	160 (73.7)	0.042	146 (71.9)	154 (72.6)	28 (73.7)	120 (72.7)	58 (74.4)	53 (79.1)	141 (78.8)	79 (78.2)	0.71
	Consume nutritious food	914 (87.6)	34 (89.5)	755 (87.8)	125 (86.2)	0.814	233 (91.7)	273 (86.7)	119 (85)	103 (88)	186 (85.7)	0.210	178 (87.7)	185 (87.3)	35 (92.1)	140 (84.8)	69 (88.5)	58 (86.6)	158 (88.3)	91 (90.1)	0.914

Table S5. Association Between Risk Perception and Sex, Age, Marital Status and Education (N=1043; %)

	Risk Perception Key Elements	Total	Sex			Age					Marital Status			Education			
			Male	Female	P-value	Early-Late Teens	Early-Late Adults	Early-Late Old	Elderly	P-value	Married	Unmarried	P-value	<=High School	Under Graduate	Graduate	P-value
B.	Risk Perception																
1	Anxiety				<0.001					0.005			0.026				0.420
	Not Anxious	49 (4.7)	33 (7.8)	16 (2.6)		14 (6.1)	29 (4.2)	5 (4.3)	1 (25.0)		29 (4.4)	20 (5.3)		6 (3.7)	25 (4.3)	18 (5.9)	
	Quite Anxious	323 (31.0)	151 (35.5)	171 (27.8)		84 (36.5)	215 (31.1)	23 (19.7)	1 (25.0)		186 (27.9)	137 (36.3)		57 (35.2)	182 (31.6)	84 (27.5)	
	Anxious	448 (43.0)	167 (39.3)	279 (45.4)		83 (36.1)	313 (45.2)	51 (43.6)	1 (25.0)		300 (45.0)	148 (39.3)		63 (38.9)	242 (42.0)	143 (46.9)	
	Very Anxious	223 (21.4)	74 (17.4)	149 (24.2)		49 (21.3)	135 (19.5)	38 (32.5)	1 (25.0)		151 (22.7)	72 (19.1)		36 (22.2)	127 (22.0)	60 (19.7)	
2	Newness				0.833					0.249			0.95				0.002
	Emerging disease	791 (75.8)	321 (75.5)	468 (76.1)		168 (73.0)	535 (77.3)	84 (71.8)	4 (100)		506 (76.0)	285 (75.6)		114 (70.4)	424 (73.6)	253 (83.0)	
	Emerging disease	252 (24.2)	104 (24.5)	147 (23.9)		62 (27.0)	157 (22.7)	33 (28.2)	0 (0.0)		160 (24.0)	92 (24.4)		48 (29.6)	152 (26.4)	52 (17.0)	
3	Severity				0.024					0.474			0.944				0.234
	Not severe	21 (2.0)	12 (2.8)	9 (1.5)		5 (2.2)	15 (2.2)	1 (0.9)	0 (0.0)		14 (2.1)	7 (1.9)		3 (1.9)	14 (2.4)	4 (1.3)	
	Quite severe	219 (21.0)	106 (24.9)	113 (18.4)		44 (19.1)	143 (20.7)	30 (25.6)	2 (50.0)		143 (21.5)	76 (20.2)		27 (16.7)	124 (21.5)	68 (22.3)	
	Severe	387 (37.1)	149 (35.1)	235 (38.2)		78 (33.9)	264 (38.2)	43 (36.8)	2 (50.0)		244 (36.6)	143 (37.9)		53 (32.7)	221 (38.4)	113 (37.0)	
	Very severe	416 (39.9)	158 (37.2)	258 (42)		103 (44.8)	270 (39.0)	43 (36.8)	0 (0.0)		265 (39.8)	151 (40.1)		79 (48.8)	217 (37.7)	120 (39.3)	
4	Infectiousness				0.007					0.516			0.078				0.010
	Slightly infectious	7 (0.7)	6 (1.4)	1 (0.2)		2 (0.9)	4 (0.6)	1 (0.9)	0 (0.0)		5 (0.8)	2 (0.5)		1 (0.6)	5 (0.9)	1 (0.3)	
	Infectious	139 (13.3)	67 (15.8)	72 (11.7)		39 (17)	88 (12.7)	11 (9.4)	1 (25.0)		77 (11.6)	62 (16.4)		32 (19.8)	81 (14.1)	26 (8.5)	
	Very infectious	897 (86.0)	352 (82.8)	542 (88.1)		189 (82.2)	600 (86.7)	105 (89.7)	3 (75.0)		584 (87.7)	313 (83.0)		129 (79.6)	490 (85.1)	278 (91.1)	
5	Contagiousness				0.888					<0.001			<0.001				0.001
	Very slow (within months)	2 (0.2)	1 (0.2)	1 (0.2)		2 (0.9)	0 (0.0)	0 (0.0)	0 (0.0)		0 (0.0)	2 (0.5)		1 (0.6)	1 (0.2)	0 (0.0)	
	Slow (within weeks	88 (8.4)	33 (7.8)	52 (8.5)		36 (15.7)	42 (6.1)	9 (7.7)	1 (25.0)		40 (6.0)	48 (12.7)		21 (13.0)	44 (7.6)	23 (7.5)	
	Quite fast (within days)	566 (54.3)	237 (55.8)	329 (53.5)		117 (50.9)	379 (54.8)	67 (57.3)	3 (75.0)		365 (54.8)	201 (53.3)		71 (43.8)	345 (59.9)	150 (49.2)	
	Immediately	387 (37.1)	154 (36.2)	233 (37.9)		75 (32.6)	271 (39.2)	41 (35)	0 (0.0)		261 (39.2)	126 (33.4)		69 (42.6)	186 (32.3)	132 (43.3)	
6	Seriousness				0.001					0.017			0.498				0.615
	Very serious	764 (73.3)	287 (67.5)	474 (77.1)		167 (72.6)	500 (72.3)	94 (80.3)	3 (75.0)		495 (74.3)	269 (71.4)		124 (76.5)	414 (71.9)	226 (74.1)	
	Serious	250 (24.0)	118 (27.8)	132 (21.5)		52 (22.6)	178 (25.7)	20 (17.1)	0 (0.0)		156 (23.4)	94 (24.9)		32 (19.8)	146 (25.3)	72 (23.6)	

	Risk Perception Key Elements	Total	Sex			Age					Marital Status			Education			
			Male	Female	P-value	Early-Late Teens	Early-Late Adults	Early-Late Old	Elderly	P-value	Married	Unmarried	P-value	<=High School	Under Graduate	Graduate	P-value
	Quite Serious	25 (2.4)	18 (4.2)	7 (1.1)		9 (3.9)	13 (1.9)	2 (1.7)	1 (25.0)		13 (2.0)	12 (3.2)		6 (3.7)	13 (2.3)	6 (2.0)	
	Not serious	4 (0.4)	2 (0.5)	2 (0.3)		2 (0.9)	1 (0.1)	1 (0.9)	0 (0.0)		2 (0.3)	2 (0.5)		0 (0.0)	3 (0.5)	1 (0.3)	
7	Total Cases				<0.001					0.709			0.405				0.503
	Very low	2 (0.2)	2 (0.5)	0 (0.0)		0 (0.0)	2 (0.3)	0 (0.0)	0 (0.0)		2 (0.3)	0 (0.0)		0 (0.0)	1 (0.2)	1 (0.3)	
	Low	21 (2.0)	17 (4)	4 (0.7)		3 (1.3)	16 (2.3)	2 (1.7)	0 (0.0)		14 (2.1)	7 (1.9)		1 (0.6)	10 (1.7)	10 (3.3)	
	High	264 (25.3)	132 (31.1)	129 (21)		54 (23.5)	171 (24.7)	37 (31.6)	2 (50.0)		177 (26.6)	87 (23.1)		39 (24.1)	150 (26.0)	75 (24.6)	
	Very high	756 (72.5)	274 (64.5)	482 (78.4)		173 (75.2)	503 (72.7)	78 (66.7)	2 (50.0)		473 (71.0)	283 (75.1)		122 (75.3)	415 (72.0)	219 (71.8)	

Table S6. Association Between Risk Perception and Physical Contact with Covid-19 patient, Occupation and Residence (N=1043; %)

	Risk Perception Key Elements	Total	Physical contact with COVID-19 patient				Occupation						Residency								
			**Yes**	**No**	**Not sure**	**P-value**	**Civil Servant**	**Private Company**	**Student**	**Housewife**	**Others**	**P-value**	**Jakarta**	**West Java**	**East Java**	**Central Java**	**South Sulawesi**	**Banten**	**Bali**	**Others**	**P-value**
B.	Risk Perception																				
1	Anxiety					0.668						0.012									0.085
	Not Anxious	49 (4.7)	2 (5.3)	40 (4.7)	7 (4.8)		9 (3.5)	21 (6.7)	9 (6.4)	0 (0.0)	10 (4.6)		9 (4.4)	7 (3.3)	1 (2.6)	7 (4.2)	1 (1.3)	3 (4.5)	11 (6.1)	10 (9.9)	
	Quite Anxious	323 (31.0)	13 (34.2)	275 (32)	35 (24.1)		63 (24.8)	104 (33.0)	57 (40.7)	34 (29.1)	65 (30.0)		63 (31.0)	73 (34.4)	19 (50.0)	49 (29.7)	27 (34.6)	21 (31.3)	40 (22.3)	31 (30.7)	
	Anxious	448 (43.0)	15 (39.5)	366 (42.6)	67 (46.2)		123 (48.4)	132 (41.9)	47 (33.6)	55 (47.0)	91 (41.9)		86 (42.4)	86 (40.6)	15 (39.5)	74 (44.8)	34 (43.6)	34 (50.7)	84 (46.9)	35 (34.7)	
	Very Anxious	223 (21.4)	8 (21.1)	179 (20.8)	36 (24.8)		59 (23.2)	58 (18.4)	27 (19.3)	28 (23.9)	51 (23.5)		45 (22.2)	46 (21.7)	3 (7.9)	35 (21.2)	16 (20.5)	9 (13.4)	44 (24.6)	25 (24.8)	
2	Newness					0.078						0.010									0.062
	Emerging disease	791 (75.8)	23 (60.5)	656 (76.3)	112 (77.2)		213 (83.9)	227 (72.1)	107 (76.4)	83 (70.9)	161 (74.2)		148 (72.9)	155 (73.1)	31 (81.6)	115 (69.7)	65 (83.3)	58 (86.6)	139 (77.7)	80 (79.2)	
	Long existing disease	252 (24.2)	15 (39.5)	204 (23.7)	33 (22.8)		41 (16.1)	88 (27.9)	33 (23.6)	34 (29.1)	56 (25.8)		55 (27.1)	57 (26.9)	7 (18.4)	50 (30.3)	13 (16.7)	9 (13.4)	40 (22.3)	21 (20.8)	
3	Severity					0.073						0.625									0.234
	Not severe	21 (2.0)	1 (2.6)	15 (1.7)	5 (3.4)		5 (2.0)	7 (2.2)	4 (2.9)	0 (0.0)	5 (2.3)		6 (3)	3 (1.4)	0 (0.0)	1 (0.6)	2 (2.6)	0 (0.0)	5 (2.8)	4 (4.0)	
	Quite severe	219 (21.0)	10 (26.3)	185 (21.5)	24 (16.6)		60 (23.6)	70 (22.2)	28 (20.0)	17 (14.5)	44 (20.3)		40 (19.7)	42 (19.8)	11 (28.9)	31 (18.8)	15 (19.2)	12 (17.9)	47 (26.3)	21 (20.8)	
	Severe	387 (37.1)	20 (52.6)	309 (35.9)	58 (40.0)		91 (35.8)	120 (38.1)	47 (33.6)	51 (43.6)	78 (35.9)		77 (37.9)	90 (42.5)	14 (36.8)	56 (33.9)	29 (37.2)	28 (41.8)	67 (37.4)	26 (25.7)	
	Very severe	416 (39.9)	7 (18.4)	351 (40.8)	58 (40.0)		98 (38.6)	118 (37.5)	61 (43.6)	49 (41.9)	90 (41.5)		80 (39.4)	77 (36.3)	13 (34.2)	77 (46.7)	32 (41)	27 (40.3)	60 (33.5)	50 (49.5)	
4	Infectiousness					0.797						<0.001									0.257

Risk Perception Key Elements		Total	Physical contact with COVID-19 patient				Occupation						Residency								
			**Yes**	**No**	**Not sure**	**P-value**	**Civil Servant**	**Private Company**	**Student**	**Housewife**	**Others**	**P-value**	**Jakarta**	**West Java**	**East Java**	**Central Java**	**South Sulawesi**	**Banten**	**Bali**	**Others**	**P-value**
	Slightly infectious	7 (0.7)	0 (0.0)	7 (0.8)	0 (0.0)		0 (0.0)	4 (1.3)	0 (0.0)	0 (0.0)	3 (1.4)		1 (0.5)	0 (0.0)	0 (0.0)	0 (0.0)	1 (1.3)	0 (0.0)	4 (2.2)	1 (1.0)	
	Infectious	139 (13.3)	5 (13.2)	113 (13.1)	21 (14.5)		14 (5.5)	50 (15.9)	28 (20.0)	15 (12.8)	32 (14.7)		31 (15.3)	25 (11.8)	3 (7.9)	29 (17.6)	11 (14.1)	8 (11.9)	23 (12.8)	9 (8.9)	
	Very infectious	897 (86.0)	33 (86.8)	740 (86)	124 (85.5)		240 (94.5)	261 (82.9)	112 (80.0)	102 (87.2)	182 (83.9)		171 (84.2)	187 (88.2)	35 (92.1)	136 (82.4)	66 (84.6)	59 (88.1)	152 (84.9)	91 (90.1)	
5	Contagiousness					0.039						0.016									0.030
	Very slow (within months)	2 (0.2)	0 (0.0)	2 (0.2)	0 (0.0)		0 (0.0)	0 (0.0)	1 (0.7)	0 (0.0)	1 (0.5)		0 (0.0)	0 (0.0)	0 (0.0)	0 (0.0)	0 (0.0)	0 (0.0)	2 (1.1)	0 (0.0)	
	Slow (within weeks	88 (8.4)	3 (7.9)	63 (7.3)	22 (15.2)		18 (7.1)	22 (7.0)	25 (17.9)	6 (5.1)	17 (7.8)		11 (5.4)	15 (7.1)	2 (5.3)	15 (9.1)	9 (11.5)	7 (10.4)	22 (12.3)	7 (6.9)	
	Quite fast (within days)	566 (54.3)	18 (47.4)	482 (56.0)	66 (45.5)		138 (54.3)	181 (57.5)	68 (48.6)	63 (53.8)	116 (53.5)		118 (58.1)	131 (61.8)	21 (55.3)	98 (59.4)	31 (39.7)	34 (50.7)	83 (46.4)	50 (49.5)	
	Immediately	387 (37.1)	17 (44.7)	313 (36.4)	57 (39.3)		98 (38.6)	112 (35.6)	46 (32.9)	48 (41.0)	83 (38.2)		74 (36.5)	66 (31.1)	15 (39.5)	52 (31.5)	38 (48.7)	26 (38.8)	72 (40.2)	44 (43.6)	
6	Seriousness					0.979						0.015									0.001
	Very serious	764 (73.3)	29 (76.3)	629 (73.1)	106 (73.1)		194 (76.4)	220 (69.8)	100 (71.4)	95 (81.2)	155 (71.4)		155 (76.4)	150 (70.8)	23 (60.5)	124 (75.2)	58 (74.4)	50 (74.6)	131 (73.2)	73 (72.3)	
	Serious	250 (24.0)	8 (21.1)	207 (24.1)	35 (24.1)		59 (23.2)	85 (27.0)	30 (21.4)	22 (18.8)	54 (24.9)		43 (21.2)	58 (27.4)	15 (39.5)	36 (21.8)	18 (23.1)	15 (22.4)	44 (24.6)	21 (20.8)	
	Quite Serious	25 (2.4)	1 (2.6)	20 (2.3)	4 (2.8)		1 (0.4)	9 (2.9)	8 (5.7)	0 (0.0)	7 (3.2)		5 (2.5)	4 (1.9)	0 (0.0)	5 (3)	2 (2.6)	2 (3.0)	4 (2.2)	3 (3.0)	
	Not serious	4 (0.4)	0 (0.0)	4 (0.5)	0 (0.0)		0 (0.0)	1 (0.3)	2 (1.4)	0 (0.0)	1 (0.5)		0 (0.0)	0 (0.0)	0 (0.0)	0 (0.0)	0 (0.0)	0 (0.0)	0 (0.0)	4 (4.0)	
7	Total Cases					0.348						0.243									0.127
	Very low	2 (0.2)	0 (0.0)	2 (0.2)	0 (0.0)		0 (0.0)	0 (0.0)	0 (0.0)	0 (0.0)	2 (0.9)		0 (0.0)	0 (0.0)	0 (0.0)	0 (0.0)	1 (1.3)	0 (0.0)	1 (0.6)	0 (0.0)	
	Low	21 (2.0)	2 (5.3)	19 (2.2)	0 (0.0)		5 (2.0)	8 (2.5)	1 (0.7)	3 (2.6)	4 (1.8)		7 (3.4)	3 (1.4)	0 (0.0)	1 (0.6)	2 (2.6)	1 (1.5)	2 (1.1)	5 (5.0)	
	High	264 (25.3)	12 (31.6)	216 (25.1)	36 (24.8)		65 (25.6)	88 (27.9)	29 (20.7)	23 (19.7)	59 (27.2)		50 (24.6)	58 (27.4)	7 (18.4)	35 (21.2)	21 (26.9)	17 (25.4)	58 (32.4)	18 (17.8)	
	Very high	756 (72.5)	24 (63.2)	623 (72.4)	109 (75.2)		184 (72.4)	219 (69.5)	110 (78.6)	91 (77.8)	152 (70.0)		146 (71.9)	151 (71.2)	31 (81.6)	129 (78.2)	54 (69.2)	49 (73.1)	118 (65.9)	78 (77.2)	

Table S7. Association Between Risk Tolerance and Sex, Age, Marital Status and Education (N=1043; %)

	Risk Perception Key Elements	Total	Sex			Age					Marital Status			Education			
			Male	Female	P-value	Early-Late Teens	Early-Late Adults	Early-Late Old	Elderly	P-value	Married	Unmarried	P-value	<=High School	Under Graduate	Graduate	P-value
	Risk Tolerance																
1	Preparedness				0.004					<0.001			0.057				0.161
	Very unprepared	114 (10.9)	43 (10.1)	71 (11.5)		17 (7.4)	72 (10.4)	25 (21.4)	0 (0.0)		84 (12.6)	30 (8)		24 (14.8)	62 (10.8)	28 (9.2)	
	Unprepared	335 (32.1)	119 (28.0)	216 (35.1)		85 (37.0)	211 (30.5)	39 (33.3)	0 (0.0)		212 (31.8)	123 (32.6)		61 (37.7)	172 (29.9)	102 (33.4)	
	Quite Prepared	494 (47.4)	209 (49.2)	285 (46.3)		100 (43.5)	348 (50.3)	44 (37.6)	2 (50.0)		314 (47.1)	180 (47.7)		63 (38.9)	283 (49.1)	148 (48.5)	
	Prepared	100 (9.6)	54 (12.7)	43 (7.0)		28 (12.2)	61 (8.8)	9 (7.7)	2 (50.0)		56 (8.4)	44 (11.7)		14 (8.6)	59 (10.2)	27 (8.9)	
2	Ability to control the risk				0.06					0.326			0.112				0.009
	Unable	53 (5.1)	21 (4.9)	32 (5.2)		10 (4.3)	37 (5.3)	6 (5.1)	0 (0.0)		40 (6.0)	13 (3.4)		11 (6.8)	23 (4.0)	19 (6.2)	
	Fairly able	429 (41.1)	161 (37.9)	268 (43.6)		86 (37.4)	292 (42.2)	50 (42.7)	1 (25.0)		281 (42.2)	148 (39.3)		64 (39.5)	226 (39.2)	139 (45.6)	
	Able	453 (43.4)	187 (44.0)	263 (42.8)		111 (48.3)	294 (42.5)	47 (40.2)	1 (25.0)		274 (41.1)	179 (47.5)		77 (47.5)	269 (46.7)	107 (35.1)	
	Very able	108 (10.4)	56 (13.2)	52 (8.5)		23 (10.0)	69 (10.0)	14 (12)	2 (50.0)		71 (10.7)	37 (9.8)		10 (6.2)	58 (10.1)	40 (13.1)	
3	Believe in the efficacy of control measures																
	**Hand sanitizing**				0.019					0.894			1.000				0.804
	Yes	1032 (98.9)	417 (98.1)	613 (99.7)		228 (99.1)	685 (99.0)	115 (98.3)	4 (100.0)		659 (98.9)	373 (98.9)		160 (98.8)	571 (99.1)	301 (98.7)	
	No	11 (1.1)	8 (1.9)	2 (0.3)		2 (0.9)	7 (1.0)	2 (1.7)	0 (0.0)		7 (1.1)	4 (1.1)		2 (1.2)	5 (0.9)	4 (1.3)	
	**Physical distancing**				<0.001					0.231			0.001				0.827
	Yes	983 (94.2)	383 (90.1)	597 (97.1)		215 (93.5)	649 (93.8)	115 (98.3)	4 (100.0)		640 (96.1)	343 (91.0)		151 (93.2)	544 (94.4)	288 (94.4)	
	No	60 (5.8)	42 (9.9)	18 (2.9)		15 (6.5)	43 (6.2)	2 (1.7)	0 (0.0)		26 (3.9)	34 (9.0)		11 (6.8)	32 (5.6)	17 (5.6)	
	**Wearing a facemask**				0.021					0.959			0.708				0.489
	Yes	1036 (99.3)	419 (98.6)	614 (99.8)		229 (99.6)	687 (99.3)	116 (99.1)	4 (100.0)		662 (99.4)	374 (99.2)		162 (100.0)	571 (99.1)	303 (99.3)	
	No	7 (0.7)	6 (1.4)	1 (0.2)		1 (0.4)	5 (0.7)	1 (0.9)	0 (0.0)		4 (0.6)	3 (0.8)		0 (0.0)	5 (0.9)	2 (0.7)	
	**Staying at home**				0.119					0.367			0.212				0.206
	Yes	1015 (97.6)	411 (96.7)	604 (98.2)		221 (96.1)	679 (98.1)	114 (97.4)	4 (100.0)		653 (98.0)	365 (96.8)		155 (95.7)	565 (98.1)	298 (97.7)	
	No	25 (2.4)	14 (3.3)	11 (1.8)		9 (3.9)	13 (1.9)	3 (2.6)	0 (0.0)		13 (2.0)	12 (3.2)		7 (4.3)	11 (1.9)	7 (2.3)	
4	Willingness to carry out the measure/s																
	**Hand sanitizing**				0.483					0.609			0.470				0.190
	Yes	1032 (99.2)	423 (99.5)	609 (99.0)		227 (98.7)	687 (99.3)	117 (100.0)	4 (100.0)		662 (99.4)	373 (98.9)		159 (98.1)	572 (99.3)	304 (99.6)	
	No	8	2	6		3	5	0	0		4	4		3	4	1	

Risk Perception Key Elements	Total	Sex			Age					Marital Status			Education			
		Male	Female	P-value	Early-Late Teens	Early-Late Adults	Early-Late Old	Elderly	P-value	Married	Unmarried	P-value	<=High School	Under Graduate	Graduate	P-value
	(0.8)	(0.5)	(1.0)		(1.3)	(0.7)	(0.0)	(0.0)		(0.6)	(1.1)		(1.9)	(0.7)	(0.3)	
**Physical distancing**				0.076					0.118			0.266				0.531
Yes	1016 (97.4)	409 (96.2)	604 (98.2)		219 (95.2)	679 (98.1)	114 (97.4)	4 (100.0)		652 (97.9)	364 (96.6)		156 (96.3)	561 (97.4)	299 (98.0)	
No	27 (2.6)	16 (3.8)	11 (1.8)		11 (4.8)	13 (1.2)	3 (2.6)	0 (0.0)		14 (2.1)	13 (3.4)		6 (3.7)	15 (2.6)	6 (2.0)	
**Wearing a facemask**				1.000					0.917			1.000				0.066
Yes	1042 (99.9)	425 (100.0)	614 (99.8)		230 (100.0)	691 (99.9)	117 (100.0)	4 (100.0)		665 (99.8)	377 (100.0)		161 (99.4)	576 (100.0)	305 (100.0)	
No	1 (0.1)	0 (0.0)	1 (0.2)		0 (0.0)	1 (0.1)	0 (0.0)	0 (0.0)		1 (0.2)	0 (0.0)		1 (0.6)	0 (0.0)	0 (0.0)	
**Staying at home**				0.139					0.161			0.661				<0.001
Yes	868 (83.5)	346 (81.4)	522 (84.9)		200 (87.0)	565 (81.6)	101 (86.3)	4 (100.0)		553 (83.0)	317 (84.1)		144 (88.9)	448 (77.8)	278 (91.1)	
No	172 (16.5)	79 (18.6)	93 (15.1)		30 (13.0)	127 (18.4)	16 (13.7)	0 (0.0)		113 (17.0)	60 (15.9)		18 (11.1)	128 (22.2)	27 (8.9)	

Table S8. Association Between Risk Tolerance and Physical Contact with Covid-19 patient, Occupation and Residence (N=1043; %)

	Risk Perception Key Elements	Total	Physical contact with COVID-19 patient				Occupation						Residence								
			**Yes**	**No**	**Not sure**	**P-value**	**Civil Servant**	**Private Company**	**Student**	**Housewife**	**Others**	**P-value**	**Jakarta**	**West Java**	**East Java**	**Centra l Java**	**South Sulawesi**	**Banten**	**Bali**	**Others**	**P-value**
C.	Risk Tolerance																				
1	Preparedness					0.007						0.639									<0.001
	Very unprepared	114 (10.9)	5 (13.2)	91 (10.6)	18 (12.4)		31 (12.2)	36 (11.4)	11 (7.9)	15 (12.8)	21 (9.7)		30 (14.8)	24 (11.3)	1 (2.6)	12 (7.3)	14 (17.9)	6 (9.0)	18 (10.1)	9 (8.9)	
	Unprepared	335 (32.1)	4 (10.5)	278 (32.3)	53 (36.6)		81 (31.9)	92 (29.2)	51 (36.4)	44 (37.6)	67 (30.9)		58 (28.6)	70 (33.0)	11 (28.9)	66 (40)	23 (29.5)	27 (40.3)	54 (30.2)	26 (25.7)	
	Quite Prepared	494 (47.4)	20 (52.6)	408 (47.4)	66 (45.5)		122 (48.0)	158 (50.2)	65 (46.4)	47 (40.2)	102 (47.0)		91 (44.8)	110 (51.9)	23 (60.5)	64 (38.8)	37 (47.4)	31 (46.3)	79 (44.1)	59 (58.4)	
	Prepared	100 (9.6)	9 (23.7)	83 (9.7)	8 (5.5)		20 (7.9)	29 (9.2)	13 (9.3)	11 (9.4)	27 (12.4)		24 (11.8)	8 (3.8)	3 (7.9)	23 (13.9)	4 (5.1)	3 (4.5)	28 (15.6)	7 (6.9)	
2	Ability to control the risk					0.090						0.251									0.121
	Unable	53 (5.1)	1 (2.6)	41 (4.8)	11 (7.6)		16 (6.3)	13 (4.1)	7 (5)	8 (6.8)	9 (4.1)		11 (5.4)	11 (5.2)	1 (2.6)	10 (6.1)	6 (7.7)	1 (1.5)	8 (4.5)	5 (5)	
	Fairly able	429 (41.1)	15 (39.5)	344 (40.0)	70 (48.3)		115 (45.3)	143 (45.4)	53 (37.9)	42 (35.9)	76 (35.0)		87 (42.9)	100 (47.2)	11 (28.9)	62 (37.6)	26 (33.3)	33 (49.3)	73 (40.8)	37 (36.6)	
	Able	453 (43.4)	16 (42.1)	380 (44.2)	57 (39.3)		94 (37.0)	130 (41.3)	67 (47.9)	56 (47.9)	106 (48.8)		89 (43.8)	75 (35.4)	25 (65.8)	76 (46.1)	40 (51.3)	26 (38.8)	72 (40.2)	50 (49.5)	
	Very able	108 (10.4)	6 (15.8)	95 (11.0)	7 (4.8)		29 (11.4)	29 (9.2)	13 (9.3)	11 (9.4)	26 (12.0)		16 (7.9)	26 (12.3)	1 (2.6)	17 (10.3)	6 (7.7)	7 (10.4)	26 (14.5)	9 (8.9)	
3	Believe in the efficacy of control measures																				

Risk Perception Key Elements		Total	Physical contact with COVID-19 patient				Occupation						Residence								
			**Yes**	**No**	**Not sure**	**P-value**	**Civil Servant**	**Private Company**	**Student**	**Housewife**	**Others**	**P-value**	**Jakarta**	**West Java**	**East Java**	**Central Java**	**South Sulawesi**	**Banten**	**Bali**	**Others**	**P-value**
	**Hand sanitizing**					0.012						0.477									0.572
	Yes	1032 (98.9)	36 (94.7)	854 (99.3)	142 (97.9)		249 (98.0)	312 (99.0)	139 (99.3)	117 (100.0)	215 (99.1)		201 (99.0)	209 (98.6)	38 (100.0)	164 (99.4)	77 (98.7)	67 (100.0)	178 (99.4)	98 (97.0)	
	No	11 (1.1)	2 (5.3)	6 (0.7)	3 (2.1)		5 (2.0)	3 (1.0)	1 (0.7)	0 (0.0)	2 (0.9)		2 (1.0)	3 (1.4)	0 (0.0)	1 (0.6)	1 (1.3)	0 (0.0)	1 (0.6)	3 (3.0)	
	**Physical distancing**					0.232						0.038									0.113
	Yes	983 (94.2)	34 (89.5)	815 (94.8)	134 (92.4)		246 (96.9)	288 (91.4)	131 (93.6)	114 (97.4)	204 (94.0)		194 (95.6)	196 (92.5)	38 (100.0)	155 (93.9)	74 (94.9)	66 (98.5)	170 (95.0)	90 (89.1)	
	No	60 (5.8)	4 (10.5)	45 (5.2)	11 (7.6)		8 (3.1)	27 (8.6)	9 (6.4)	3 (2.6)	13 (6.0)		9 (4.4)	16 (7.5)	0 (0.0)	10 (6.1)	4 (5.1)	1 (1.5)	9 (5.0)	11 (10.9)	
	**Wearing a facemask**					0.080						0.189									0.306
	Yes	1036 (99.3)	38 (100.0)	856(9 9.5)	142 (97.9)		253 (99.6)	310 (98.4)	140 (100.0)	117 (100.0)	216 (99.5)		199 (98.0)	211 (99.5)	38 (100.0)	164 (99.4)	77 (98.7)	67 (100.0)	179 (100.0)	101 (100.0)	
	No	7 (0.7)	0 (0.0)	4 (0.5)	3 (2.1)		1 (0.4)	5 (1.6)	0 (0.0)	0 (0.0)	1 (0.5)		4 (2.0)	1 (0.5)	0 (0.0)	1 (0.6)	1 (1.3)	0 (0.0)	0 (0.0)	0 (0.0)	
	**Staying at home**					0.328						0.702									0.655
	Yes	1015 (97.6)	37 (97.4)	842 (97.9)	139 (95.9)		248 (97.6)	308 (97.8)	135 (96.4)	116 (99.1)	211 (97.2)		199 (98.0)	209 (98.6)	38 (100)	161 (97.6)	76 (97.4)	64 (95.5)	172 (96.1)	99 (98.0)	
	No	25 (2.4)	1 (2.6)	18 (2.1)	6 (4.1)		6 (2.4)	7 (2.2)	5 (3.6)	1 (0.9)	6 (2.8)		4 (2.0)	3 (1.4)	0 (0.0)	4 (2.4)	2 (2.6)	3 (4.5)	7 (3.9)	2 (2.0)	
4	Willingness to carry out the measure/s																				
	**Hand sanitizing**					0.242						0.367									0.200
	Yes	1032 (99.2)	37 (97.4)	855 (99.4)	143 (98.6)		253 (99.6)	313 (99.4)	137 (97.9)	116 (99.1)	216 (99.5)		199 (98.0)	211 (99.5)	38 (100.0)	165 (100.0)	77 (98.7)	67 (100.0)	179 (100.0)	99 (98.0)	
	No	8 (0.8)	1 (2.6)	5 (0.6)	2 (1.4)		1 (0.4)	2 (0.6)	3 (2.1)	1 (0.9)	1 (0.5)		4 (2.0)	1 (0.5)	0 (0.0)	0 (0.0)	1 (1.3)	0 (0.0)	0 (0.0)	2 (2.0)	
	**Physical distancing**					0.779						0.043									0.375
	Yes	1016 (97.4)	37 (97.4)	839 (97.6)	140 (96.6)		249 (98.0)	305 (96.8)	132 (94.3)	117 (100.0)	213 (98.2)		199 (98.0)	205 (96.7)	35 (92.1)	160 (97.0)	76 (97.4)	67 (100.0)	176 (98.3)	98 (97.0)	
	No	27 (2.6)	1 (2.6)	21 (2.4)	5 (3.4)		5 (2.0)	10 (3.2)	8 (5.7)	0 (0.0)	4 (1.8)		4 (2.0)	7 (3.3)	3 (7.9)	5 (3.0)	2 (2.6)	0 (0.0)	3 (1.7)	3 (3.0)	
	**Wearing a facemask**					0.899						0.094									0.089
	Yes	1042 (99.9)	38 (100.0)	859 (99.9)	145 (100. 0)		254 (100.0)	315 (100.0)	140 (100.0)	116 (99.1)	217 (100.0)		203 (100.0)	212 (100. 0)	38 (100.0)	165 (100.0)	77 (98.7)	67 (100.0)	179 (100.0)	101 (100.0)	
	No	1 (0.1)	0 (0.0)	1 (0.1)	0 (0.0)		0 (0.0)	0 (0.0)	0 (0.0)	1 (0.9)	0 (0.0)		0 (0.0)	0 (0.0)	0 (0.0)	0 (0.0)	1 (1.3)	0 (0.0)	0 (0.0)	0 (0.0)	
	**Staying at home**					<0.001						<0.001									0.485
	Yes	868 (83.5)	24 (63.2)	747 (86.9)	99 (68.3)		211 (83.1)	234 (74.3)	133 (95)	105 (89.7)	187 (86.2)		169 (83.3)	175 (82.5)	30 (78.9)	136 (82.4)	72 (92.3)	58 (86.6)	149 (83.2)	81 (80.2)	
	No	172 (16.5)	14 (36.8)	113 (13.1)	46 (31.7)		43 (16.9)	81 (25.7)	7 (5)	12 (10.3)	30 (13.8)		34 (16.7)	37 (17.5)	8 (21.1)	29 (17.6)	6 (7.7)	9 (13.4)	30 (16.8)	20 (19.8)	
